# Navigating Perioperative Challenges in Pott’s Spine: A Comprehensive Review

**DOI:** 10.7759/cureus.59871

**Published:** 2024-05-08

**Authors:** Souvik Banik, Sheetal Madavi

**Affiliations:** 1 Anaesthesiology, Jawaharlal Nehru Medical College, Datta Meghe Institute of Higher Education and Research, Wardha, IND

**Keywords:** pott’s spine, tuberculosis, multidisciplinary approach, surgical management, perioperative challenges, tuberculous spondylitis

## Abstract

Pott’s spine, or tuberculous spondylitis, remains a significant public health concern in regions where tuberculosis is endemic. The management of Pott’s spine poses unique perioperative challenges due to the complexity of the disease process, including vertebral destruction, spinal instability, and neurological compromise. This comprehensive review explores the intricacies of navigating perioperative challenges in Pott’s spine surgery. Beginning with an overview of Pott’s spine, including its etiology, clinical presentation, and classification, the review delves into the significance of perioperative challenges in this condition. Emphasis is placed on the need for multidisciplinary collaboration, meticulous preoperative assessment, and tailored surgical planning to optimize outcomes while minimizing the risk of complications. Critical considerations in the preoperative, intraoperative, and postoperative phases of care are discussed in detail, including patient assessment, imaging modalities, surgical techniques, anesthesia considerations, and postoperative rehabilitation. Special considerations such as pediatric Pott’s spine and multidrug-resistant tuberculosis are also addressed. The review concludes by summarizing key points, highlighting implications for clinical practice, and providing recommendations for future research. By synthesizing current evidence and clinical expertise, this review offers valuable insights into the optimal management of perioperative challenges in Pott’s spine, ultimately aiming to improve patient outcomes and reduce the burden of this debilitating condition.

## Introduction and background

Pott’s spine, also known as tuberculous spondylitis, is a form of spinal tuberculosis named after Sir Percivall Pott, who first described it in the 18th century [1]. It is a destructive form of tuberculosis affecting the vertebral column, primarily caused by *Mycobacterium tuberculosis*. Pott’s spine typically involves the thoracic and lumbar vertebrae and can lead to vertebral collapse, spinal deformity, and neurological deficits if left untreated [2].

Perioperative management of Pott’s spine poses significant challenges due to the complex nature of the disease. Surgical intervention is often required to prevent or correct spinal deformity, decompress neural structures, and eradicate the infection [3]. However, the presence of active tuberculosis, spinal instability, neurologic deficits, and systemic comorbidities complicates the perioperative course. Additionally, the risk of postoperative complications, including recurrence of infection, neurological deterioration, and instrumentation failure, necessitates careful planning and management [4].

This comprehensive review explores the perioperative challenges of managing Pott’s spine. Through an in-depth analysis of current literature and clinical practices, this review seeks to provide insights into the optimal strategies for navigating these challenges. Key aspects to be addressed include preoperative assessment and planning, intraoperative decision-making, postoperative care, and long-term outcomes. By elucidating the intricacies of perioperative management, this review aims to contribute to the enhancement of patient outcomes and the advancement of clinical practice in the field of spinal tuberculosis.

## Review

Understanding Pott’s spine

Etiology and Pathogenesis

**Figure 1:**
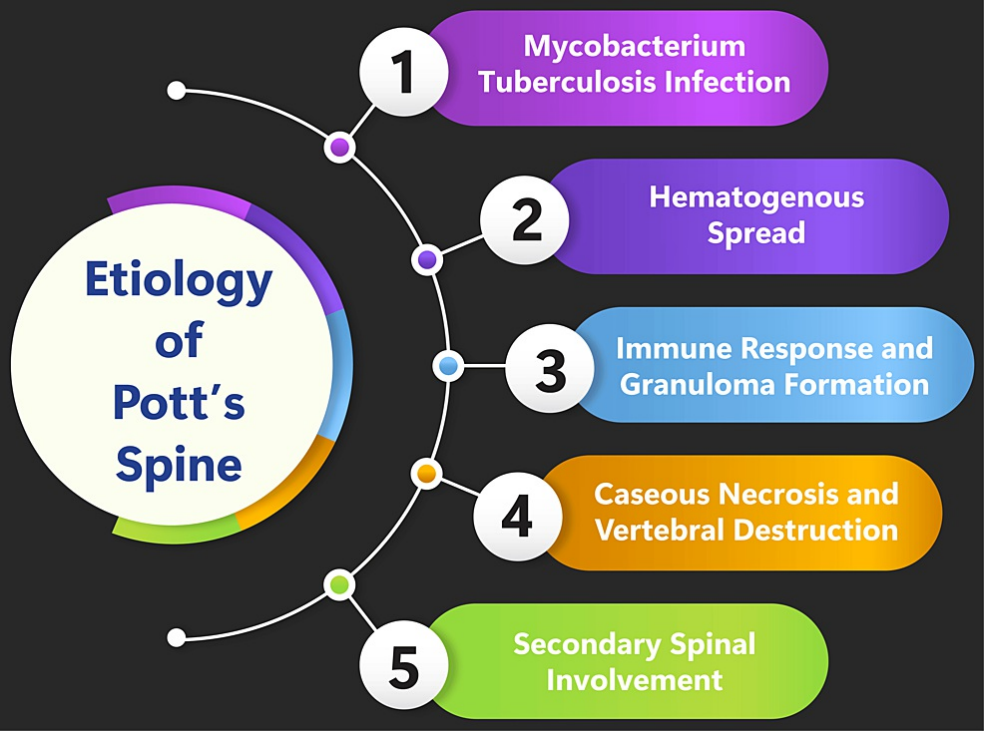
Etiology of Pott’s disease. Image credit: Dr. Souvik Banik.

The etiology and pathogenesis of Pott’s disease involve the hematogenous spread of tuberculous infection to the cancellous bone of the vertebral body, typically originating from a primary source of infection in either the pulmonary or genitourinary site [2,5,6]. The spread of infection occurs through a rich vascular plexus in the sub-chondral region of each vertebra, facilitated by the anterior and posterior spinal arteries, leading to the involvement of multiple contiguous vertebrae [3,7]. In classic spinal tuberculosis, the infection initially affects the anterior aspect of the vertebral body and then spreads to the adjacent intervertebral discs. Children are commonly affected in the intervertebral discs due to high vascularity, while adults or older people are more commonly affected in the vertebral bodies due to age-related avascularity [3,7]. The infection destroys the intervertebral disc space and adjacent vertebral bodies, leading to the collapse of spinal elements, anterior wedging, and characteristic angulation and gibbus formation, which is a palpable deformity due to the involvement of multiple vertebrae [3,7]. Moreover, the destruction of the disc space and vertebral bodies can result in spinal deformity, with kyphosis being more prominent in the thoracic spine due to anterior collapse, potentially leading to spinal cord compression and paraplegia secondary to the narrowing of the spinal canal [3,7]. The compression of motor fibers in anterior spinal tuberculosis and posterior spinal tuberculosis can affect motor function due to the susceptibility of motor fibers to pressure and ischemia, respectively [3,7]. The etiology of Pott’s disease is shown in Figure 1.

Clinical Presentation and Diagnosis

Pott’s disease, also known as spinal tuberculosis or tuberculous spondylitis, results from the spread of *Mycobacterium tuberculosis* to the spine. Its clinical presentation typically manifests as chronic back pain, stiffness, and various neurological deficits, including spinal cord compression, paraplegia, paresis, impaired sensation, nerve root pain, and cauda equina syndrome [8-10]. While the disease can affect any part of the spine, the thoracic and lumbar segments are most commonly involved, constituting 80% to 90% of cases [9,10]. Diagnosis of Pott’s disease primarily relies on imaging modalities such as plain radiographs, computed tomography (CT), and magnetic resonance imaging (MRI) [10]. MRI, in particular, is regarded as the gold-standard diagnostic tool for spinal tuberculosis due to its superior sensitivity in detecting features such as disc collapse/destruction, cold abscesses, vertebral wedging/collapse, marrow edema, and spinal deformities [10]. Complementary to MRI, CT scanning offers detailed bony information, while ultrasound and CT-guided needle aspiration or biopsy facilitate early microbiological diagnosis [10]. The natural progression of Pott’s disease involves hematogenous spread leading to spinal involvement. It can result in various neurological deficits, with stages of Pott’s paraplegia classified based on motor and sensory impairments [9]. Children, owing to the immaturity and flexibility of their spines, are particularly susceptible to severe deformities, necessitating vigilant monitoring until skeletal maturity [9]. Treatment for Pott’s disease aims to eradicate the infectious agent, prevent or manage spinal deformities, and address neurological complications and the rehabilitation process. This typically involves a combination of chemotherapy targeting *Mycobacterium tuberculosis* and orthopedic interventions to manage spinal column issues [10]. While modern treatment strategies have reduced the prevalence of Pott’s disease in developed countries, it remains a significant health concern in less developed regions, disproportionately affecting children [10].

Classification and Staging

Pott’s disease, a prevalent granulomatous spine infection caused by tubercle bacilli, accounts for 50-60% of cases [11]. It advances through several stages, beginning with the implantation or incipient stage, followed by the early destruction stage, advanced destruction and collapse stage, neurological involvement stage, and, finally, the residual deformity and aftermath stage [12]. During the early destruction stage, typically lasting two to four months, discernible features include diminished disc space, paradiscal erosion, and kyphosis of fewer than 10 degrees [12]. Subsequently, the advanced destruction and collapse stage, occurring between three and nine months, witnesses extensive vertebral damage, potentially leading to spinal cord compression and paraplegia [12]. Paraplegia in Pott’s disease is further categorized into early and late paraplegia based on the onset timing and symptom severity [12]. Early paraplegia manifests during the active tuberculosis infection phase, predominantly observed in adults within two years of infection onset. In contrast, late paraplegia emerges in patients with healed tuberculosis, typically defined as neurological symptoms appearing after two years of primary infection [12]. The most useful classification system for Pott’s paraplegia, involving spinal cord involvement, is the modified version of Tuli’s classification, comprising five stages [13]. While encompassing the majority of neurologic abnormalities, this classification system provides a comprehensive framework for understanding disease progression and associated neurologic deficits in Pott’s disease [13]. Classifications and staging systems for Pott’s spine are presented in Table 1.

**Table 1:** Classifications and staging systems for Pott’s spine.

Classification	Stages
Tuli’s classification	I. Incipient or implantation stage
	II. Early destruction stage
	III. Advanced destruction and collapse stage
	IV. Neurological involvement stage
	V. Residual deformity and aftermath stage
Modified Tuli’s classification for Pott’s paraplegia	1. Backache, no neurological deficit
	2. Mild neurological deficit (sensory)
	3. Gross neurological deficit (motor weakness)
	4. Complete paraplegia with recoverable sphincter disturbance
	5. Complete paraplegia with irrecoverable sphincter disturbance

Preoperative considerations

Patient Assessment and Selection

Before proceeding with surgery for Pott’s disease, a thorough assessment of the patient’s general health is imperative. This assessment evaluates the patient’s overall health status, including nutritional status, the presence of anemia, and the impact of tuberculosis on organ function. Such evaluation aids in determining the patient’s suitability for surgery and guides preoperative optimization [11]. Concurrent evaluation and treatment of active pulmonary tuberculosis, if present, is essential before surgery to mitigate the risk of infection spread during the procedure [11]. Additionally, careful consideration of potential drug interactions between antituberculous therapy (ATT) and anesthetic agents is crucial to avoid adverse effects during surgery [11]. Given the prevalence of autonomic dysfunction in patients with spinal tuberculosis, assessing for underlying autonomic dysfunction is prudent. Understanding the implications of autonomic dysfunction on anesthesia and perioperative management ensures comprehensive care [11]. Diagnostic imaging studies, including CT myelography and MRI, play a pivotal role in assessing the extent of spinal cord compression, deformity, and vertebral destruction. These imaging modalities provide essential information to guide surgical planning and decision-making [14]. Determining the surgical indications is paramount and hinges on various factors such as the extent of vertebral destruction, the presence of cord compression, spinal deformity, and instability. Surgical intervention may be warranted in cases of severe abscess compression, neurologic deficits, or involvement of the cervical spine [14]. Establishing a comprehensive plan for postoperative follow-up is vital to monitor the patient’s response to therapy, medication compliance, and any signs of disease progression or complications. Long-term follow-up is particularly crucial in children to monitor growth potential and in older patients for late-onset complications [14]. Incorporating consultations from orthopedic surgeons, neurosurgeons, and rehabilitation teams into the patient’s care ensures a multidisciplinary approach, facilitating comprehensive management and optimal outcomes [14].

Imaging Modalities and Interpretation

MRI is the premier diagnostic tool for assessing Pott’s spine due to its remarkable sensitivity in detecting spinal tuberculosis. MRI reveals various pathological features, including disc involvement, subligamentous spread of abscesses, vertebral body collapse, and large abscess collections characterized by thin abscess walls [10,15,16]. CT complements MRI by providing detailed bony information essential for diagnosis. CT scans offer visualization of irregular lytic lesions, sclerosis, disc collapse, and disruption of bone circumference. Furthermore, they excel in delineating soft tissues, particularly in epidural and paraspinal regions. CT imaging is particularly effective in defining the shape and calcification of soft tissue abscesses, with calcification being a common feature in tuberculous lesions [6]. Radionuclide studies, employing techniques such as bone scintigraphy with 99mTc-Diphosphonates and 67Ga-citrate, serve to differentiate infectious from non-infectious diseases, assess metabolic rates of lesions, detect multifocality, and identify active disease that may be unresponsive to treatment. While offering comprehensive whole-body evaluations, these techniques have limitations in differentiating between pyogenic and non-pyogenic infections [15]. Positron emission tomography-computed tomography (PET-CT) imaging, primarily utilized in oncologic assessments, demonstrates diagnostic capabilities comparable to MRI in detecting spondylodiscitis, including Pott’s disease. PET-CT provides detailed insights into metabolic activity, facilitating diagnosis and treatment monitoring [6].

Multidisciplinary Approach to Treatment Planning

The multidisciplinary approach to treatment planning involves diverse disciplines in crafting or refining a comprehensive management strategy for a particular disease. This collaborative process fosters improved communication, consistency, and direction among treatment team members, ultimately leading to more effective patient care [17-19]. Key steps in developing a multidisciplinary treatment plan typically involve assessment, problem identification, planning (including setting priorities, establishing long- and short-term goals, and determining interventions), and evaluation [18]. In healthcare settings, employing a multidisciplinary approach is essential for addressing complex patient needs, particularly in cases involving conditions such as substance use disorders, mental health issues, cancer care, and implant restorations [19,20]. By harnessing the expertise of professionals from various fields, including medical doctors, nurses, addiction counselors, psychiatrists, and others, this approach ensures the development of a holistic and personalized treatment plan tailored to the individual’s unique requirements [20]. The advantages of a multidisciplinary treatment plan go beyond addressing the primary condition; it also considers co-occurring disorders, physical and mental health issues, and overall well-being. This comprehensive approach facilitates the creation of an integrated care pathway that optimizes patient outcomes and supports long-term recovery [17-19].

Preoperative Optimization Strategies

Surgical planning for Pott’s spine involves meticulous attention to detail, encompassing thorough debridement of tuberculosis infection lesions, standardized and effective debridement techniques, and adequate spinal stabilization [21,22]. Preoperative imaging is crucial in evaluating the disease’s extent and determining the surgical approach. Imaging modalities such as X-ray, CT, and MRI provide valuable information regarding lesion location and severity, spinal cord compression, and any associated deformities [21,22]. Optimizing the patient’s medical condition before surgery is paramount to minimize postoperative complications. This includes addressing nutritional deficiencies, correcting anemia, and managing comorbidities such as diabetes or hypertension [21,22]. Preoperative ATT is essential for disease control and reducing the risk of postoperative complications. The duration of ATT before surgery should be individualized based on the patient’s clinical and radiological response to treatment [21,22]. A thorough assessment of neurological function preoperatively guides surgical planning and predicts the risk of postoperative neurological complications. This involves evaluating muscle strength, sensory function, and reflexes [21,22]. Given the potential for significant blood loss during Pott’s spine surgery, preoperative optimization of blood loss is crucial. This includes assessing coagulation status, correcting abnormalities, and optimizing blood volume and hemoglobin levels [21,22]. Assessing pulmonary function preoperatively is essential to mitigate the risk of pulmonary complications associated with surgery. Evaluating lung function and optimizing respiratory status are critical components of preoperative care [21,22]. Similarly, preoperative assessment of pain helps tailor the surgical approach and anticipate postoperative pain management needs. This includes evaluating pain intensity, location, quality, and response to analgesics [23,24]. Understanding patients’ expectations and preferences before surgery is essential to ensure informed decision-making and alignment with surgical goals [23,24]. Assessing social support preoperatively helps determine if the patient has adequate support during the postoperative period, including assessing living arrangements, caregiver availability, and access to postoperative care [23,24].

Intraoperative management

Surgical Approaches and Techniques

Surgical management of Pott’s spine encompasses various approaches and techniques, including anterior, posterior, and combined approaches. The anterior approach is most suitable for patients lacking involvement of posterior vertebral structures, facilitating direct access to the lesion, optimal visualization, and complete spinal cord decompression [21]. Conversely, the posterior approach offers superior posterior stabilization benefits [21]. Employing a one-stage posterior circumferential fusion can circumvent the need for thoracotomy or thoracoabdominal procedures, which may impose detrimental stress on the lungs, especially in elderly or frail patients [21]. Minimally invasive surgery presents a newer option, either as a standalone approach or in conjunction with open procedures [25]. Intraoperative management entails various considerations, including the use of antifibrinolytics such as tranexamic acid (TXA) and aminocaproic acid to mitigate blood loss, evaluating concomitant active pulmonary tuberculosis, the patient’s overall health status, potential drug interactions between ATT and anesthetic agents, underlying autonomic dysfunction, concerns regarding one-lung ventilation during thoracoscopic spine surgery, and the risk of nosocomial infection transmission [11]. Effective postoperative pain management is essential and can be achieved through local wound infiltration with ropivacaine, adrenaline, and dexmedetomidine, which has demonstrated improved pain control in Pott’s spine patients [11]. However, it is crucial to remain vigilant for spinal epidural abscess, a neurological emergency that demands prompt diagnosis and immediate surgical spinal cord decompression [11].

Anesthesia Considerations

Anesthesia considerations in Pott’s spine surgery are pivotal owing to the distinctive challenges posed by the disease. Anesthesiologists play a crucial role in the perioperative management of patients with spinal tuberculosis undergoing either spine or non-spine surgeries [11]. Specific anesthesia considerations encompass various aspects, including the imperative to avoid exacerbating neurological deterioration during maneuvers in cervical spine surgery. Additionally, early diagnosis facilitated by CT myelography and MRI is essential to address potential neurologic complications. Furthermore, employing local wound infiltration with ropivacaine, adrenaline, and dexmedetomidine aids in effective postoperative pain management for patients with tubercular spine conditions [11]. In pregnant patients with Pott’s spine undergoing spine surgery in the prone position, anesthesia management becomes even more complex as it must address both obstetric and surgical concerns [26]. Antepartum surgical management of Pott’s paraplegia entails several considerations, such as preoperative assessment of the fetal heart rate, implementing anti-aspiration prophylaxis measures, continuous monitoring of vital signs, and careful positioning to mitigate complications related to aortocaval compression and autonomic dysfunction [26].

Intraoperative Monitoring and Navigation

Intraoperative monitoring and navigation are pivotal aspects of perioperative management in Pott’s spine surgery. Intraoperative neurophysiological monitoring (IONM) comprises a series of diagnostic modalities to prevent perioperative injury to the spinal cord and nerve roots during spine procedures [27]. This monitoring is conducted by neurophysiologists, trained specialists who collaborate with the surgical team to create and record electrical signals within the nervous system, closely linked to motor and sensory function [27]. Throughout the operation, electromyography and evoked potentials are continuously monitored, alerting surgeons to waveform alterations and breaches of specific alarm criteria [27]. A multimodality approach to spinal cord monitoring is deemed the most effective utilization of IONM [27]. In Pott’s spine surgery, intraoperative monitoring encompasses the use of somatosensory evoked potentials and motor-evoked potentials for continuous assessment of spinal cord function [11]. These techniques enable ongoing evaluation of spinal cord function, which is critical due to the inherent risk of spinal cord injury in Pott’s spine surgery. However, continuous monitoring of these techniques is not feasible, and interpretation can be challenging in patients with underlying motor disorders. Additionally, these techniques are sensitive to anesthetic agents, such as inhalational agents and neuromuscular blockers [11]. Intraoperative navigation and robotics also play essential roles in perioperative management for Pott’s spine surgery. These technologies facilitate precise placement of implants and reduction of spinal deformities, crucial considerations given the risk of spinal cord injury inherent in Pott’s spine surgery [28].

Management of Intraoperative Complications

Intraoperative complications in Pott’s spine surgery demand a multifaceted approach for effective management. This encompasses several strategies, including ensuring proper patient positioning, employing antifibrinolytics such as TXA and aminocaproic acid to mitigate blood loss, and meticulously addressing concomitant active pulmonary TB [11]. Furthermore, the classical triad of presentation in Pott’s spine, i.e., intense localized back pain, progressive neurologic deficit, and fever, underscores the importance of prompt diagnosis through CT myelography and MRI, followed by immediate surgical spinal cord decompression when warranted [11]. Intraoperative management should prioritize disease eradication, preventing or correcting spine deformities, and effective management of neurologic complications [11]. Surgical intervention in Pott’s spine aims for thorough debridement, spinal cord decompression, and robust spinal stabilization to achieve favorable clinical and radiological outcomes [29]. Additionally, it is crucial to factor in the patient’s overall health status, potential drug interactions between ATT and anesthetic agents, autonomic dysfunction, and the risk of nosocomial infection transmission during Pott’s spine surgery [30].

Postoperative care

Early Postoperative Period: Pain Management and Mobilization

The early postoperative phase is important for effective pain management, and mobilization is crucial in enhancing recovery and minimizing complications. The research underscores that insufficient postoperative analgesia can impede mobilization, underscoring the necessity of managing pain effectively to facilitate early mobilization post-surgery [31,32]. Early mobilization following gastrointestinal surgery has been associated with a spectrum of benefits, including hastened gastrointestinal recovery, diminished postoperative pain intensity, improved sleep quality, and reduced incidence of fatigue [33]. Moreover, early mobilization protocols have been correlated with shorter hospital stays in certain studies, highlighting the favorable impact of mobilization on postoperative outcomes [33]. In postoperative pain management, providing adequate analgesia is paramount for patient comfort and fostering early mobilization, a cornerstone of enhanced recovery after surgery initiatives [34,35]. Various analgesic techniques, such as multimodal or balanced analgesia, have been deployed to achieve optimal pain control and facilitate early mobilization during the postoperative period [32,35]. Research indicates that robust postoperative pain management enhances patient comfort and satisfaction, resulting in earlier mobilization, reduced incidences of pulmonary and cardiac complications, diminished risk of deep vein thrombosis, accelerated recovery, and lowered healthcare expenses [35].

Complication Prevention and Management

Prevention and management of perioperative complications represent pivotal facets of care across various surgical procedures. Achieving success in this realm demands high surgical expertise and rigorous standardization of techniques [36]. Central to this endeavor are the imperatives of prevention, early detection, and judicious management, particularly for patients harboring functional risk factors [37]. Given the potential for severe complications and their consequential impact on long-term survival, meticulous attention to perioperative complication prevention and management is paramount [37]. In procedures such as laparoscopic transperitoneal radical prostatectomy and endoscopic extraperitoneal radical prostatectomy, complication rates typically range between 2% and 17%, encompassing rare but plausible occurrences such as vascular injuries, bowel injury, lymphocele formation, port-site hernia, anastomotic leakage, gas embolism, and catheter obstruction [36]. Effective prevention and management of these complications hinge upon adept surgical proficiency and robust standardization of techniques [36]. In esophagectomy, where the stakes are equally high, attention to perioperative complication prevention and management is paramount for procedural success. Preoperative cardiological assessment is critical for patients with cardiac comorbidities, with necessary medications such as β-blockers administered to mitigate stress reactions and forestall perioperative cardiac complications, particularly supraventricular tachyarrhythmias [37]. Moreover, preoperative identification and clarification of alcohol withdrawal syndrome in individuals with alcohol abuse are essential to preempt potential postoperative challenges [37]. Preoperative risk analysis emerges as a linchpin in preventing perioperative complications, with general and specialized classification systems available to assess individual patient risk before surgery [37]. Leveraging data from extensive databases, an individual multiplicator derived from diverse organ functions can be utilized to gauge the overall risk profile of the patient [37]. Subsequently, high-risk patients should be carefully evaluated for suitability for surgery, while those deemed to have a moderate risk profile should undergo optimal preoperative preparation [37]. In minimally invasive surgery, meticulous attention should be paid to techniques such as the closure of larger vessels with paperclips and thorough inspection of port sites post-trocar removal to preempt bleeding incidents [37]. Given the direct correlation between surgical blood loss and mortality, bleeding prevention assumes paramount importance [37]. Equally crucial is the prompt detection and appropriate management of postoperative complications, necessitating vigilant clinical oversight by experienced surgeons and intensivists, supported by robust diagnostic equipment, to ensure early intervention and resolution [37].

Rehabilitation and Physiotherapy

Rehabilitation and physiotherapy constitute integral components in the comprehensive management of Pott’s spine, a manifestation of spinal tuberculosis. Post-surgery, patients typically embark on a physiotherapy regimen spanning at least a month to rectify structural deformities and forestall further complications [38]. This rehabilitative approach holds the potential to alleviate pain, bolster respiratory function, enhance sensory capabilities, and foster overall functional independence among individuals grappling with Pott’s disease [39]. A tailored physiotherapy program is pivotal in optimizing patient outcomes, encompassing a spectrum of interventions such as mobility exercises, lower limb and core musculature strengthening, breathing techniques, and postural correction exercises [39]. Additionally, sensory re-education assumes a crucial role in the rehabilitation of individuals afflicted with Pott’s disease [39]. Evidence suggests that diverse modalities, including transcutaneous electrical neuromuscular stimulation, aquatic therapy, overground training (walking program), aerobic exercise, and trunk strengthening interventions, can yield noteworthy reductions in pain, psychological distress, and disability among patients with Pott’s disease [40]. Moreover, physiotherapy management holds particular significance in the post-spinal decompression surgery and post-spinal fusion surgery phases of Pott’s spine treatment. Interventions such as spinal stabilization exercises, Maitland techniques, back school programs, and targeted exercise and strengthening interventions have demonstrated notable efficacy in improving global outcomes for individuals grappling with Pott’s disease [40].

Long-Term Follow-up and Monitoring

Long-term follow-up and monitoring are imperative for individuals afflicted with Pott’s disease, a variant of spinal tuberculosis. Following the completion of tuberculosis treatment, it is customary for children who have recovered from spinal tuberculosis to undergo annual follow-up visits at the hospital until they reach skeletal maturity [41]. This proactive approach stems from the heightened risk of deformity progression even years after the resolution of the infection in pediatric cases, necessitating ongoing monitoring to track skeletal development [40]. However, despite being a standard protocol, a recent study revealed a significant gap between recommended practice and real-world implementation, with a striking 84% of children diagnosed with spinal tuberculosis failing to receive the prescribed follow-up care [41]. This discrepancy underscores the urgent need for improved adherence to follow-up guidelines to safeguard optimal outcomes for patients grappling with Pott’s disease. In addition to consistent follow-up, individuals with Pott’s disease may benefit from ongoing physiotherapy interventions to address any residual neurological deficits or spinal deformities. Evidence suggests that aerobic exercise, physical therapy, and trunk-strengthening interventions can yield tangible improvements by reducing pain, mitigating psychological distress, and enhancing functional capacity in patients with Pott’s disease [40]. This holistic approach to long-term management underscores the importance of comprehensive care in optimizing outcomes and enhancing the quality of life for individuals affected by Pott’s disease.

Future directions and innovations

Advances in Surgical Techniques and Technology

Integrating robotic arms into surgical procedures represents a significant advancement known as robotic-assisted surgery. This innovation enhances surgical precision and visualization and minimizes invasiveness, ultimately leading to shorter recovery times and improved patient outcomes. Surgeons and patients increasingly embrace this cutting-edge approach, heralding a new era of advanced surgical techniques [42]. Augmented reality (AR) and virtual reality (VR) technologies offer transformative possibilities in healthcare, as seen in the utilization of AR and VR. AR enhances surgical procedures by overlaying real-time holographic images and data, improving surgeon efficiency and clinical outcomes. Conversely, VR is a valuable tool for surgical training and simulation, allowing surgeons to hone their skills and refine techniques in a virtual environment before operating on actual patients [42]. Artificial Intelligence (AI) is revolutionizing surgical technology through its integration into various procedures, as observed in AI in surgery. Advanced algorithms assist clinicians in tasks ranging from robotic surgery to precise cancer detection, enhancing surgical precision and accuracy. AI’s incorporation into medical practice reshapes surgical procedures across different specialties, benefiting practitioners and patients [43]. Adopting three-dimensional (3D) imaging techniques, such as CT and MRI, transforms surgical care into 3D Imaging and telepresence surgery. These imaging modalities enable surgeons to visualize patients’ anatomy in three dimensions, facilitating precise preoperative planning and intraoperative navigation. Furthermore, telepresence surgery expands surgeons’ capabilities beyond traditional limitations, enhancing surgical outcomes by providing an extended reach and enhanced visualization [44].

Pharmacological Innovations

One innovative approach involves the utilization of 3D-printed scaffolds incorporating drugs such as isoniazid (INH) and rifampicin (RIF) to mitigate drug resistance in osteoarticular tuberculosis debridement procedures [45]. These bioengineered delivery systems, exemplified by 3D-printed tablets with discrete compartmentalization for RIF and INH, hold promise in reducing drug degradation and enhancing combination treatment efficacy [45]. By employing bilayered tablets containing both INH and RIF within a 3D-printed scaffold, the release site can be modulated using pH-sensitive polymers to mitigate pH-dependent deterioration and bolster therapeutic effectiveness [45]. Targeted therapy emerges as another avenue of innovation, leveraging biocompatible polymers such as polylactic glycolic acid to develop controlled anti-tubercular drug (ATD) delivery systems [45]. For instance, surface functionalization of drug carriers with ligands recognized by macrophage receptors enables tissue-resident macrophage targeting, enhancing ATD delivery efficiency [45]. Additionally, nanotechnology presents vast potential in crafting novel and targeted delivery systems, exemplified by transforming growth factor-β1-specific siRNA nanoliposomes loaded with INH, RIF, and pyrazinamide, offering promise in improving spinal tuberculosis chemotherapy [45]. Furthermore, nanotechnology facilitates the development of inhaled or orally delivered nanocarriers for extended ATD release, thereby enhancing patient adherence [45]. Prodrugs with low aqueous solubility can be harnessed to formulate long-acting therapeutics, inhibiting rapid dissolution and enabling slow elimination from the body, thus necessitating lower drug doses for injection [45]. Notably, drugs such as bedaquiline, boasting a longer half-life, higher lipophilicity, and lower minimum inhibitory concentration for *Mycobacterium tuberculosis*, are ideal candidates for incorporation into long-acting injectable formulations [45]. A one-time, large-dose, controlled-release delivery system in the gastrointestinal tract offers numerous advantages over conventional injectable depot formulations, including simplified administration and reduced risk of immunologic reactions [45].

## Conclusions

Navigating the perioperative challenges inherent in Pott’s spine surgery requires a comprehensive approach that encompasses preoperative assessment, meticulous surgical planning, intraoperative vigilance, and thorough postoperative care. This review has underscored the significance of accurate diagnosis, multidisciplinary collaboration, and adherence to evidence-based guidelines in optimizing patient outcomes. Moving forward, clinicians must integrate these insights into their clinical practice, emphasizing the importance of a multidisciplinary team, evidence-based protocols, and ongoing education. Furthermore, there is a pressing need for continued research to enhance our understanding of Pott’s spine further, refine treatment strategies, and evaluate the efficacy of emerging interventions. By addressing these priorities, clinicians can strive to improve the care and outcomes of patients affected by this complex condition.
